# Predicting Thermal Adaptation by Looking Into Populations’ Genomic Past

**DOI:** 10.3389/fgene.2020.564515

**Published:** 2020-09-25

**Authors:** Andrés J. Cortés, Felipe López-Hernández, Daniela Osorio-Rodriguez

**Affiliations:** ^1^Corporación Colombiana de Investigación Agropecuaria AGROSAVIA, C.I. La Selva, Rionegro, Colombia; ^2^Departamento de Ciencias Forestales, Facultad de Ciencias Agrarias, Universidad Nacional de Colombia – Sede Medellín, Medellín, Colombia; ^3^Division of Geological and Planetary Sciences, California Institute of Technology (Caltech), Pasadena, CA, United States

**Keywords:** coalescent theory, genome-wide association studies, genome-wide selection scans, genome–environment associations, phylogeography, breeder’s equation, genomic prediction, machine learning

## Abstract

Molecular evolution offers an insightful theory to interpret the genomic consequences of thermal adaptation to previous events of climate change beyond range shifts. However, disentangling often mixed footprints of selective and demographic processes from those due to lineage sorting, recombination rate variation, and genomic constrains is not trivial. Therefore, here we condense current and historical population genomic tools to study thermal adaptation and outline key developments (genomic prediction, machine learning) that might assist their utilization for improving forecasts of populations’ responses to thermal variation. We start by summarizing how recent thermal-driven selective and demographic responses can be inferred by coalescent methods and in turn how quantitative genetic theory offers suitable multi-trait predictions over a few generations via the breeder’s equation. We later assume that enough generations have passed as to display genomic signatures of divergent selection to thermal variation and describe how these footprints can be reconstructed using genome-wide association and selection scans or, alternatively, may be used for forward prediction over multiple generations under an infinitesimal genomic prediction model. Finally, we move deeper in time to comprehend the genomic consequences of thermal shifts at an evolutionary time scale by relying on phylogeographic approaches that allow for reticulate evolution and ecological parapatric speciation, and end by envisioning the potential of modern machine learning techniques to better inform long-term predictions. We conclude that foreseeing future thermal adaptive responses requires bridging the multiple spatial scales of historical and predictive environmental change research under modern cohesive approaches such as genomic prediction and machine learning frameworks.

## On the Challenges of Studying Genomic Thermal Adaptation

Warming is imposing an unprecedented climate emergency on nature, food, energy supply, and economy around the world (Ripple et al., 2020). While evolutionary genomics may improve prediction of populations’ responses to thermal change (Waldvogel et al., 2020a), geologic records of temperature and carbon dioxide (CO_2_) variations (Supplementary Figure S1) are also insightful into the coupling of biodiversity, climate, and the carbon cycle and hence may help predicting the consequences of future carbon emissions (Zachos et al., 2008). For instance, several reports of fire activity (Whitlock and Bartlein, 2003; Bush et al., 2008) and hydroclimate changes (Wang et al., 2017) as records of thermal changes during the Holocene have taught us that extinction is a slow process and that many species may already be functionally extinct (Cronk, 2016). A key modern advance has precisely been to couple the extinction risk with the migratory potential under an ecological niche conservatism scenario (Steinbauer et al., 2018), and predictions of population-level genomic and phenotypic responses to thermal change (Hoffmann and Sgro, 2011). Although atmospheric CO_2_ has been found to be better correlated with richness of (plant) species (Supplementary Figure S1C) than temperature itself throughout the Cenozoic up until 20 Mya (Jaramillo et al., 2006; Royer and Chernoff, 2013), we need to improve our understanding on how thermal change vulnerability impacts current and historical adaptive genetic variation in order to enhance populations response projections (Razgour et al., 2019).

**FIGURE 1:**
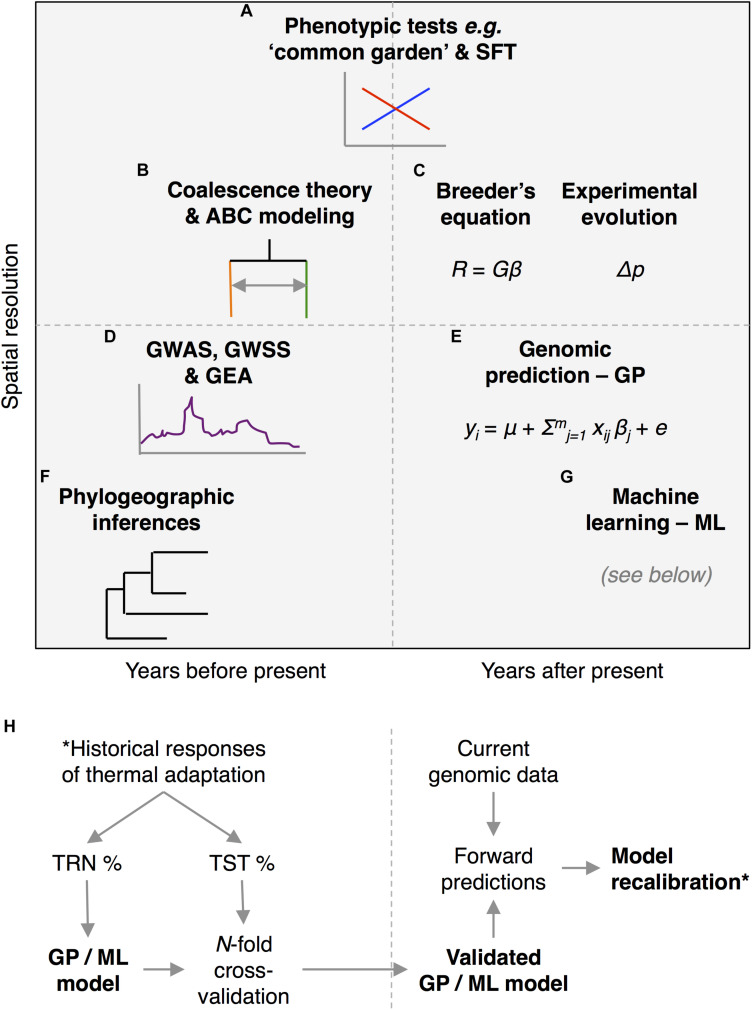
Potential approaches to assess populations’ thermal adaptation by looking into their genomic past. Genomic analyses allow reconstructing populations’ adaptive responses to previous events of climate change across various temporal scales **(A,B,D,F)**, as a tool to improve forecasting **(C,E,G,H)**. **(A)** Empirical approaches such as replicated “common garden” (provenance) tests and space-for-time (SFT) substitution allow studying *in situ* ongoing genomic thermal adaptation. The inset plot exemplifies a significant genotype-by-environment (*G*x*E*) interaction, as can be quantified using reciprocal transplant experiments between habitat types that differ in their thermal stress. **(B)** Coalescent and approximate Bayesian computation (ABC) analyses help infer recent thermal-driven selective and demographic responses. The inset diagram shows a typical coalescent genealogy depicting divergence with gene flow. **(C)** The breeder’s equation predicts responses of genetically correlated traits over one generation (vector *R*) given standardized selection gradients to thermal stress (vector β) by means of the variance–covariance matrix (*G*) of additive genetic parameter estimates. Alternatively, experimental evolution traces real-time changes in allele frequencies (Δ*p*) across generations. **(D)** When genomic signatures of thermal selection are under divergent selection after several generations, genome-wide association (GWAS), and selection (GWSS) scans, as well as genome–environment associations (GEA), allow characterizing the genomic architecture of thermal adaptation. The inset Manhattan plot schematizes a hypothetical genomic scan between populations that contrast in their thermal adaptation. **(E)** Modern high-throughput genotyping may facilitate predictions of the thermal adaptive potential over multiple generations using infinitesimal models under a genomic prediction (GP) framework. **(F)** Phylogeographic approaches offer an understanding of the genomic consequences of deep-time thermal shifts at an evolutionary time scale. The inset tree represents an imaginary phylogeny. Finally, **(G)** machine learning (ML) approaches **(H)** trained using heterogeneous past responses to thermal variation may enhance long-term predictions of the thermal adaptive potential. ML’s *modus operandi*, as GPs, requires partitioning the calibrating historical dataset between training (TRN) and testing (TST) subsets that are iteratively imputed into a *N*-fold cross-validation scheme.

Genomes are diverse in signatures of the populations’ evolutionary past across timescales (Wolf and Ellegren, 2017) and therefore are informative on historical adaptive responses to ancient and more recent events of climate change (Figure 1 and Table 1). By revealing the nature of these signatures and learning from previous reactions to environmental change, genomics can truly assist modern predictions aimed at incorporating responses beyond migration. Yet, disentangling often confused selective and demographic signatures from those due to genetic drift and genomic constrains is challenging (Ellegren and Galtier, 2016), consequently delaying the factual utilization of genomics for forecasting. Therefore, in this mini-review we envision summarizing modern tools from the genomic era that are enriching our comprehension of the genetic consequences of past and recent climate change, while offering a perspective on how to improve predictive models that incorporate thermal adaptation. Specifically, we aim prospecting how genomic prediction (GP) and machine learning (ML) approaches may offer cohesive frameworks to (1) integrate more traditional, but heterogeneous, genomic, and ecological datasets across temporal scales, by (2) maximizing prediction accuracies, while (3) understating the relative contribution of the underlying genomic processes. This is still a future avenue of research, and so we close by offering perspectives. Different drivers of the genomic landscape to thermal adaption (Gompert et al., 2014; Ravinet et al., 2017; Cortés and Blair, 2018; López-Hernández and Cortés, 2019), such as disruptive and background selection, gene flow (Miller et al., 2020), shared ancestral polymorphism, and mutation/recombination rate variation (Feder et al., 2012; Ellegren and Wolf, 2017; Cortés et al., 2018b), have been identified. In order to discern among them, a first necessary step toward the evaluation of the adaptive potential involves typifying the genomic landscape by using summary statistics like nucleotide diversity, π (Nei, 1987), and relative, F_*ST*_ (Weir and Cockerham, 1984), and absolute, D_*XY*_ (Nei, 1987), divergence. F_*ST*_ vs. D_*XY*_ contrasts inform population divergence in the presence of gene flow (co-occurrence of peaks in both profiles), recurrent selection across subpopulations (F_*ST*_ peaks match shallow D_*XY*_ valleys), and selective sweeps predating the subpopulations’ split (F_*ST*_ peaks match deep D_*XY*_ valleys) (Nachman and Payseur, 2012; Cruickshank and Hahn, 2014; Irwin et al., 2016). Inferences are more robust if carried out across replicated samplings of contrasting populations (e.g., in terms of thermal variation) within a hierarchically nested framework of divergence (Cortés et al., 2018b). A second step refers to the detection of selection signatures, if any – i.e., hard vs. soft selection sweeps (Pritchard et al., 2010; Zahn and Purnell, 2016), which must be followed by a third validation step across replicated demographics (Roesti et al., 2014; Lotterhos and Whitlock, 2015) and temporal levels (Nosil and Feder, 2011; Matos et al., 2015; Fragata et al., 2018).

**TABLE 1:** Case studies that have addressed thermal adaptation at different temporal scales using diverse genetic analyses.

Analytical approach	Diagram	Data sources	Main finding	References
Coalescence theory and ancestry distribution models	Figure 1B	20 alpine plant species across the European Alps genotyped with AFLP markers and analyzed with ancestry distribution models	Ancestry distribution models open new perspectives to forecast population genetic changes within species	Jay et al., 2012
Coalescence theory in a SFT framework	Figure 1B	273 *Salix* genets in 12 SFT populations genotyped with 7 SSRs	There is asymmetric gene flow across a thermal gradient that may be affected under future climate conditions	Cortés et al., 2014
Coalescence theory	Figure 1B	Exome re-sequencing of 48 *Populus trichocarpa* individuals	Effective population size has varied in concert with atmospheric temperature deviation from the past c. 120,000 years	Zhou et al., 2014
Quantitative genetics	Figure 1C	Review of models on whether evolutionary changes within species can contribute to species adapting to global thermal change	Evolutionary processes and trait trade-offs (*Q* matrix) need to be incorporated into schemes that try to manage thermal impacts	Hoffmann and Sgro, 2011
Quantitative genetics	Figure 1C	Review discussing thermal adaptation to climate change from an evolutionary physiological perspective	Species’ physiological, genetic and plastic (Nicotra et al., 2010) capacities can aid in forecasting their response to thermal change	Chown et al., 2010
Quantitative genetics	Figure 1C	Physiological model that simulates thermal tolerance assays for multilocus quantitative traits in *D. melanogaster*	Realized heritabilities of knockdown temperature may underestimate the true heritability of the upper thermal limit	Rezende et al., 2010; Santos et al., 2012
Breeder’s equation in 2-habitats SFT design	Figure 1C	1,061 *Salix herbacea* genotypes, from 2 habitats in a SFT design, screened for 6 thermally influenced traits and 7 SSRs	Significant heritable variation in morphology and phenology might help *S. herbacea* adapt to thermal stress	Sedlacek et al., 2016
Quantitative genetics and breeder’s equation	Figure 1C	166 lines of *D. melanogaster* assessed for cold tolerance at 5 temperatures	Low thermal tolerance is environment specific and evolvability decreases with increasing developmental temperatures	Ørsted et al., 2019
Quantitative genetics and breeder’s equation	Figure 1C	4,267 25- to 35-year-old European larch trees growing in 21 reforestation installations across 4 distinct climatic regions in Austria	Genetic evaluation across broad thermal gradients permits delineation of suitable reforestation areas under future climates	Lstiburek et al., 2020
GWAS	Figure 1D	Review on molecular-level regulation of the annual growth cycle in temperate and boreal regions	Merging genomic analyses with more quantitative approaches will aid studies on how species cope with thermal changes	Singh et al., 2017
eGWAS	Figure 1D	Whole-genome transcriptional responses in *D. subobscura* subjected to threefold replicated laboratory thermal shocks	Many genes appear to be involved in thermal adaptation, as expected for the adaptive evolution of a complex trait	Laayouni et al., 2007
GWAS across a SFT latitudinal gradient	Figure 1D	446 *Populus trichocarpa* trees from a latitudinal gradient screened for bud-break in 2 provenance trials and with 2.2-M SNPs	Variation in bud-break reflects differential selection for thermal functions likely to be affected by climate warming	McKown et al., 2018
GWSS across a SFT latitudinal gradient	Figure 1D	Two populations of *D. subobscura* from different latitudes introduced to a new common laboratory environment and WGS	Populations followed different genetic routes to reach predictable and similar adaptive phenotypic outcomes	Seabra et al., 2017
GWSS given a modern heat wave	Figure 1D	Long-term time series of seasonal genetic data in *D. subobscura*	Genetic constitution of the populations transiently shifted to summer-like frequencies during the 2011 heat wave	Rodriguez-Trelles et al., 2013
GWSS in 2 postglacial lineages	Figure 1D	48 *Populus alba* ramets from 2 postglacial recolonization lineages genotyped with GWS for 1.7-M SNP markers	Selection from standing variation implies the potential for rapid evolution of *P. alba* populations in the face of thermal change	Stölting et al., 2015
GEA at a continental scale	Figure 1D	78 Andean and Mesoamerican wild bean accessions with 23,373 GBS-derived SNPs and 3 bioclimatic heat stress indices	24 associated loci with contrasting habitat types flank 22 heat shock protein genes (Simões et al., 2003; Sørensen et al., 2003)	López-Hernández and Cortés, 2019
GEA at a latitudinal gradient	Figure 1D	Four populations of *D. subobscura* from different latitudes screened for 4 candidate loci for thermal adaptation in inversions	Inversion frequency clines are being maintained by local thermal adaptation in face of gene flow	Simões and Pascual, 2018
GEA at a regional scale	Figure 1D	79 natural *Fagus sylvatica* populations, 144 SNPs out of 52 thermal candidate genes, and 87 environmental predictors	*F. sylvatica* exhibits local genetic adaptation to thermal heterogeneity at the regional scale (Swiss Alps)	Pluess et al., 2016
GEA at a regional scale	Figure 1D	140 wild tomato accessions, 6,830 SNPs, and redundancy analysis (RDA), structural equation modeling (SEM), and generalized dissimilarity modeling (GDM)	Regional differences in the abiotic environment contribute to genomic divergence within a wild tomato species	Gibson and Moyle, 2020
Genomic prediction (GP)	Figure 1E	48 cows genotypes with a BovineLD BeadChip and studied in climate-controlled chambers that simulate a heat wave event	GP for heat tolerance may increase resilience and welfare in animal breeding to increased incidence and duration of heat events	Garner et al., 2016
Backward genomic prediction (GP)	Figure 1E	Re-sequencing of 15 1900-year-old maize cobs from Turkey Pen Shelter, and GBS data of 1,316 modern landraces for training	Thermal adaptation drove modern maize divergence and was selected *in situ* from ancient standing variation 2000 years ago	Swarts et al., 2017
Genomic prediction (GP)	Figure 1E	287 elite spring wheat lines assessed in a 90K Illumina array for traits as thermal time to flowering in 18 heat/drought environments	GP is capable to predict complex traits and find the best environments to adapt new crop lines to heat and drought stress events	Sukumaran et al., 2017
Genomic prediction (GP)	Figure 1E	3,485 wheat lines genotyped with 9,285 GBS-derived SNPs and phenotyped for grain yield in heat and drought environments	GP can be used to increase the size of plant nurseries by considering un-phenotyped lines for heat and drought stress-resilience	Juliana et al., 2019
Fossil record	Figure 1F	Palynological neotropical plant diversity of 1,411 morpho-species and 287,736 occurrences (65–20 million years ago)	Low Paleocene flora diversity, more diverse early Eocene flora exceeding Holocene levels, and a decline at early Oligocene	Jaramillo et al., 2006
Phylogenetics	Figure 1F	Thoreau’s dataset of the Concord (MA) flora that provides data on changes in species abundance and flowering time (150 years)	Thermal change has shaped the phylo-genetically biased pattern of species loss in species that do not respond to temperature	Willis et al., 2008
Fossil record	Figure 1F	Pollen and macroscopic charcoal from the Erazo profile (Ecuador)	Global Pleistocene temperature change can radically alter vegetation communities on the Andean flank in western Amazonia	Cardenas et al., 2011
Phylogeographic inferences – fossils	Figure 1F	Long-term ecological records and their relevance to climate change predictions for a warmer world	Range shifts, community turnover, genetic adaptation, and an increase in diversity are observed during warmer intervals	Willis and MacDonald, 2011
Phylogeographic inferences	Figure 1F	17 time-calibrated phylogenies of major tetrapod clades and climatic data from distributions of > 500 extant species	Rates of projected climate change dramatically exceed past rates of thermal niche evolution among vertebrate species	Quintero and Wiens, 2013
Phylogeographic inferences	Figure 1F	Niche shifts among populations within 56 plant and animal species using time-calibrated phylogenetic trees	Rates of change in thermal niches in plant and animal populations have been much slower than projected climate change	Jezkova and Wiens, 2016
Phylogenetic-assisted modeling	Figure 1F	9,737 records for 1,312 plant species and phylogenetic correlation matrix as an additional random effect	Tropical plants do not have narrower heat tolerances, but are more at risk due to their upper thermal limits (Feeley et al., 2020)	Sentinella et al., 2020
Dynamic eco-evolutionary modeling	Figure 1G	Four endemic Alpine plant species analyzed with niche modeling, and individual-based demographic and genetic simulations	Monitoring species’ local abundance instead of their range better informs on species’ extinction risks under thermal change	Cotto et al., 2017
Machine learning (ML)	Figure 1G	Species geographic distributions modeling using maximum entropy (MaxEnt)	ML modeling can be used for discrimination of suitable vs. unsuitable areas for the species with presence-only datasets	Phillips et al., 2017
Machine learning (ML)	Figure 1G	Temporal uncertainty framework to assess when and where cultivation of key crops in sub-Saharan Africa will become unviable	Incremental, preparatory and transformational adaptation phases enable projected crop transformational changes	Rippke et al., 2016
Machine learning (ML)	Figure 1G	Random forest in Himalaya’s *Betula* for last inter-glaciation, present (1970–2000) and future (2061–2080) conditions	Biodiversity in high elevation ecosystems is sensitive to global environmental changes, especially temperature warming	Mohapatra et al., 2019
Machine learning (ML)	Figure 1G	Modeling of the spatiotemporal distribution in the present and the future of pine in heat scenarios (RCP 4.5 y RCP 8.5) by MaxEnt	There were good predictions for both climate change scenarios, and two contrasted tendencies of progressive evolution	Garah and Bentouati, 2019
Machine learning (ML)	Figure 1G	Association between gene expression and critical temperature in divergent trout populations was measured by random forest	The “gradient boosting” approach showed that evolution for higher upper thermal tolerance is possible	Chen et al., 2018
Machine learning (ML) + phylogenetic diversity	Figure 1G	Predictive models of taxonomic and phylogenetic diversity using vascular plant database for the United States	Native phylogenetic diversity is likely to decrease over the next half century despite increases in species richness	Park et al., 2020
The potential of big data	Figure 1G	Special issue inspired by the symposium “Fitness landscapes, big data, and the predictability of evolution”	Understanding evolutionary adaptive responses in the face of epistasis is a major need that could benefit from big data	Visser et al., 2018
Genomic prediction (GP) + machine learning (ML)	Figures 1E,G	*ca.* 11,000 wheat landrace accessions assessed for 40,000 GBS-derived SNPs and traits possibly related with heat stress	Deep learning should be integrated with GBLUP for the study of complex traits and the *G*x*E* interaction	Montesinos-Lopez et al., 2018
Genomic prediction (GP) + machine learning (ML)	Figures 1E,G	*ca.* 3,500 wheat landrace accessions examined for 2,038 GBS-derived SNPs in 4 environments of drought and 2 of heat stress	MLP and SVM were competitive in genomic prediction of complex traits possibly related to heat stress as days to heading	Montesinos-Lopez et al., 2019

*Examples enlighten how analytical approaches that try to reconstruct populations’ past genetic adaptive responses to previous events of climate change could be proxies for better forecasting. This compilation is built for illustrative purposes and is not meant to be exhaustive. Examples are sorted as in Figure 1. SFT, space-for-time substitution, GWAS, genome-wide association study; eGWAS, expression GWAS; GWSS, genome-wide selection scans; GEA, genome–environment associations; SSRs, simple sequence repeats; SNP, single-nucleotide polymorphism; WGS, whole-genome sequencing; GBS, genotyping-by-sequencing; SVM, support vector machine; MLP, multilayer perceptron; GP, genomic prediction; ML, machine learning.*

Exclusively phenotypic empirical methods (Figure 1A), such as *in situ* monitoring, growth chamber experiments, and “common garden” (provenance) tests (Miller et al., 2020), constitute baseline evidence of thermal adaptation and should therefore inform more advanced genomic approaches. Naturally available environmental gradients (e.g., elevation or latitudinal clines) can also be used as proxies for climate change (Wheeler et al., 2016; Cortés and Wheeler, 2018), which is known as space-for-time (SFT) substitution. Replicated “common garden” tests (a.k.a. reciprocal transplants) carried out in an SFT framework are in turn useful to test whether populations can cope with changes through local adaptation (standing variation) or via phenotypic plasticity, especially in long-living species (Bridle and Vines, 2007; Sedlacek et al., 2015). Within an SFT framework, restricted gene flow can lead to small-scale genetic structures (Stanton et al., 1997) or distorted source/sink-like patterns (e.g., Cortés et al., 2014) driven by environmental factors (Nathan and Muller-Landau, 2000). Asymmetric migratory potential in a local scale may provide suitable habitats within only a few meters of the current locations (Yamagishi et al., 2005; Scherrer and Körner, 2011) but may also lead to narrowly adapted populations, even in the face of gene flow (Fitzpatrick et al., 2015), that may respond poorly to future conditions (North et al., 2011; Miller et al., 2020).

## From Recent Genetic Responses to Short-Term Predictions

### Coalescence Informs on Contemporary Thermal-Driven Selective and Demographic Changes

In order to trace back thermal-driven selective and demographic changes at recent temporal scales (Figure 1B), coalescent theory (Wakeley, 2008) helps in discriminating among authentic signatures of selection and those related to demography (e.g., bottlenecks and among populations reduced gene flow), from spurious covariates (Yeaman and Otto, 2011) such as lineage sorting (Wolf and Ellegren, 2017; Becher et al., 2020) and inversions (Dolgova et al., 2010; Fragata et al., 2014). Recursive simulation-based tools to incorporate the mutation/selection balance (Bustamante et al., 2001) across various scenarios of divergence and gene flow are approximate Bayesian computation – ABC (Csilléry et al., 2010; Cornuet et al., 2014), and pairwise sequentially Markovian coalescent – PSMC (Nadachowska-Brzyska et al., 2016). These approaches can inform how isolated populations that usually occupy climates with scarce habitat complexity (Flantua et al., 2019) may favor thermal generalists, while intricate local-scale heterogeneity at larger scales could trigger (Hughes, 2006; Cortés et al., 2018a) thermal specialists with limited migration potential (Cuesta et al., 2019). They can also model population sizes (Beerli, 2006) in concert with thermal changes (Zhou et al., 2014; Lehnert et al., 2019). Yet, these approaches may be limited by computational burden as they rely on simulation-based rejection sampling, while much effort is gone into the design of multiple scenarios, dimensionality reduction, and feature selection (Schrider and Kern, 2018).

### The Breeder’s Equation Assists Multi-Trait Predictions Over a Few Generations

In order for thermal adaptation to happen, there must be heritable trait variation upon which selection, enforced by climate change, acts (Darwin, 1874). A simple deterministic model that condenses this evolutionary paradigm, aiding in the forecast of adaptive trait responses across few generations, comes from the quantitative genetic discipline and is known as the breeder’s equation (Figure 1C). Its multivariate form (Walsh, 2008) allows predicting responses of genetically correlated traits (vector *R*) to standardized thermal selection gradients (vector β) over one generation, so that *R* = *G*β, where *G* is the variance–covariance matrix of additive genetic parameter estimates – a proxy for traits’ heritabilities and trade-offs (Falconer and Mackay, 1996). The potential evolutionary response can therefore be computed using selection-gradient estimates derived from fitness proxies (i.e., fitness values regressed as a function of standardized trait values) and marker-based heritabilities (Lynch and Ritland, 1999). This approach by itself is not novel, but what makes it powerful is that it can be coupled with SFT (Wheeler et al., 2014), among other trials, to predict thermal responses to thermal change (Sedlacek et al., 2016). Yet, a major drawback is that selection gradients heavily depend on the nature of the fitness proxies (Sedlacek et al., 2016). Alternatively, experimental evolution studies (Exposito-Alonso et al., 2019) could test more explicitly how rapidly growing populations may respond to different thermal scenarios (Kawecki et al., 2012) that, together with evolve and re-sequence analyses (Turner and Miller, 2012), may contribute to understand the genetic basis of short-term thermal adaptation.

## From Deeper Genomic Signatures of Selection to Mid-Term Predictions

### Genome-Wide Scans Reveal Signatures of Divergent Selection to Past Thermal Adaptation

Assuming that enough generations have passed as to exhibit divergent selection to thermal changes, genome-wide association (GWAS) (Hirschhorn and Daly, 2005) and selection (GWSS) (Sabeti et al., 2007) scans (Figure 1D) are essential analytical tools to reconstruct the genomic architecture of adaptive trait divergence to thermal stress (Lecheta et al., 2020; Zwoinska et al., 2020). These methods assume that some allele variants are in linkage disequilibrium (LD) (Slatkin, 2008) with causal variants that influence the adaptive phenotype (Morris and Borevitz, 2011; Tam et al., 2019), a.k.a. genetic “hitchhiking” (Maynard Smith and Haigh, 1974; Feder and Nosil, 2010). An interface between GWAS and GWSS studies where *loci* are directly correlated with niche’s thermal variables is named genome–environment association (GEA) (Forester et al., 2016) and is insightful to infer past thermal adaptation, too (Hancock et al., 2011; Pluess et al., 2016; López-Hernández and Cortés, 2019). Yet, these approaches partly disregard non-additive and highly polygenic architectures (Stephan, 2016; Csillery et al., 2018; Barghi et al., 2020) and may be misleading (Maher, 2008; Pennisi, 2014) if standardized data (Waldvogel et al., 2020b) and statistical covariates (Lambert and Black, 2012), such as population stratification (Barton et al., 2019) and genomic constrains (Wray et al., 2013; Huber et al., 2016), are incorrectly accounted for.

### Genomic Prediction May Assist Forecasting of Adaptive Traits Over Multiple Generations

A cutting-edge development that materialized after bringing genomics into quantitative genetics theory is genomic prediction (GP) (Desta and Ortiz, 2014; Crossa et al., 2017; Grattapaglia et al., 2018). GP uses historical phenotypic data to adjust marker-based infinitesimal (Figure 1E) models (Meuwissen et al., 2001; Gianola et al., 2006; de los Campos et al., 2013) that may overcome some of the restraints described in the previous section. GP may offer a more thoughtful picture of complex traits (e.g., thermal adaptation), presumably regulated by many low-effect *loci* (Pritchard et al., 2010). GP has so far informed predictions of single adaptive traits in populations with known pedigrees (Saint Pierre et al., 2012; Cros et al., 2019) and hybrid origins (Technow et al., 2014; Tan et al., 2017), as well as multi-trait inferences across diverse unrelated populations (Crossa et al., 2007, 2016; Resende et al., 2012; Suontama et al., 2019) under genotype by environment interactions (GxE) (Montesinos-Lopez et al., 2018; Crossa et al., 2019) facing polygenic climate adaptation (Isabel et al., 2020). GP of thermal adaptive traits across multiple generations and populations may be incipient (Table 1), yet it harbors a promising potential, as was demonstrated by reversely predicting unobserved thermal phenology in 1900-year-old ancient corn (Swarts et al., 2017), and as we prospect in the last section of this mini-review.

## From Deep-Time Genomic Consequences of Thermal Shifts to Long-Term Predictions

### Phylogeography Offers Insights Into Past Responses at an Evolutionary Scale

Phylogeographic inferences (Figure 1F) offer insights into how species (1) diversify (Quintero and Wiens, 2013) and (2) face the effects of past thermal variation (Jezkova and Wiens, 2016; Richardson et al., 2019) by boosting complex interactions such as species facilitation (Wheeler et al., 2015), adaptive introgression, and hybrid speciation (Coyne and Orr, 2004; Abbott et al., 2013; Payseur and Rieseberg, 2016; Marques et al., 2019). For instance, interspecific hybrids with intermediate niche requirements may rescue population’s gene pools in the face of climate change, while they can also display signals of heterosis for thermal adaption due to dominance on recessive alleles or overdominance via novel allele combinations (Abdelmula et al., 1999; Leinonen et al., 2011). Modern phylogeographic inferences currently rely on abundant and unlinked genetic markers (Bryant et al., 2012) that are capable of bypassing traditional assumptions of single gene mutation models (Caliebe, 2008) while accounting for scenarios of reticulate evolution (Vargas et al., 2017). Marker-based inferences also offer higher resolution to validate cases where adaptive radiation (Madriñán et al., 2013), and ecological parapatric speciation resulted from local patterns of environmental variation (Cortés et al., 2018a) that may resemble those expected by thermal change. Mosaics of local-habitat heterogeneity can ultimately enforce thermal pre-adaptation (Cortés and Wheeler, 2018). Distance-based phylogenic reconstruction without proper out-groups (Baum et al., 2005; Cortés, 2013) is yet a major risk of these approaches.

### Machine Learning May Bridge Historical Genomics and Long-Term Predictions

A promising way to simultaneously make sense of multiple sources of historical genomic data that can be utilized to predict populations’ adaptive responses is by merging them into a machine learning (ML) framework (Figures 1G,H). ML bypasses the “curse of dimensionality” and benefits from high-dimensional inputs of heterogeneous dependent variables (“features”) without *a priori* knowledge of their joint probability distribution (Schrider and Kern, 2018). This improves predictions’ “recall” (true positive) rate among a set of possible responses, especially when the classification is iteratively trained using “labeled” data (i.e., historical thermal responses may offer novel calibration datasets, Table 1) via *N*-fold cross-validation. ML has been routinely used to make ecological niche modeling (Phillips et al., 2017; Valencia et al., 2020) and functional predictions across genomes (Libbrecht and Noble, 2015). Yet, ML may likely displace other tools useful to characterize the genomic consequences of thermal adaptation, already introduced in this mini-review, such as ABC modeling (Liu et al., 2019) and GWSS (Schrider and Kern, 2018).

## Concluding Remarks

Thermal adaptation is a complex polygenic trait well-described in terms of its genetic architecture and selection footprints across a wide range of phylogenetically diverse taxa (Way and Oren, 2010; Valladares et al., 2014; López-Hernández and Cortés, 2019). While genomics has enabled these achievements that rely on past events of thermal variation, forward predictions remain one step behind partly because (1) disentangling selective and demographic drivers of the genomic landscape from fortuitous genomic constrains (Logan and Cox, 2020) is puzzling (Ellegren and Galtier, 2016) and (2) merging these heterogeneous signatures and data sources into a cohesive predictive framework was unfeasible, until recently. In this mini-review, we advocated for novel approaches that may enhance our understanding of the genetic consequences of past climate change, while offering new avenues to calibrate more accurate predictive models of the thermal adaptive potential. For instance, ML advances are likely to now move beyond species distribution modeling (Phillips et al., 2017) and functional genomics (Libbrecht and Noble, 2015) to permeate the backward interpretation of recent genetic demographic responses and genomic signatures to historical thermal selection by updating popular but sometimes intractable methods such as ABC modeling and GWSS (Schrider and Kern, 2018). Meanwhile, GP and ML might boost forward predictions of the adaptive potential beyond a single generation by training multifactorial models that can try incorporating genomic heterogeneous evidence of historical thermal adaption across a wide spectrum of temporal scales. Ultimately, understanding how biotas formed in response to historical environmental change may improve our ability to predict and mitigate the threats to species posed by global warming (Ding et al., 2020).

Despite GP’s and ML’s being useful to comprehend and predict thermal adaptation, these new paradigms are not exempt of criticism. A reiterative misconception is that because these methodologies aim at strengthening predictions and classification boundaries, they do not offer a mechanistic understanding of the subjacent processes. However, even though GP and ML rely on algorithmically generated models, both are far from “black boxes” because they allow direct measurement of the contribution of each genetic marker (Resende et al., 2012; Spindel et al., 2016) and “feature” (Schrider and Kern, 2018), to the point that they can offer higher resolution than traditional genetic mapping (Hirschhorn and Daly, 2005) and deterministic model building (Otto and Day, 2007) techniques. A second misconception assumes computational burden. Although both GP and ML require a large number of simulations, they do not depend on rejection sampling, which means they may efficiently use all of the simulations to inform the mapping of historical thermal data to parameters (Schrider and Kern, 2018).

## Future Directions

So far, GP and ML have been mostly utilized to address thermal adaptation individually (Table 1). For instance, GP has been used to project heat tolerance in diverse wheat lines (Sukumaran et al., 2017; Juliana et al., 2019), and bovine genotypes (Garner et al., 2016), in all cases more as a proof of concept. Similarly, ML approaches have not only deepened our understating on populations’ range shifts in the light of thermal variation (Rippke et al., 2016; Garah and Bentouati, 2019; Mohapatra et al., 2019) but also assisted eGWAS of critical temperature thresholds (Chen et al., 2018) and phylogenetic forecasting in plants (Park et al., 2020). However, since GP and ML are both cutting-edge tools, there is still room and need for new developments. For instance, merging more cohesively past adaptive responses to previous events of environmental change into cutting-edge analytical frameworks like GP and ML will ultimately allow predicting whether populations’ adaptive potential may keep up with the pace of current thermal increase (Franks and Hoffmann, 2012; Franks et al., 2014). Swarts et al. (2017) illustrates that across-temporal predictions may be useful not only to improve forecasting (Sweet et al., 2019) but also to better understand previous responses to thermal variation, since they used backward GP to demonstrate that thermal adaptation in maize was selected *in situ* from ancient standing variation 2000 years ago. By enlightening on the nature of these historical genetic signatures to past climate change, genomics can also enhance predictions that aim at incorporating adaptive responses beyond extirpation and range shifts (Chen et al., 2011).

Data sources incorporated into GP and ML can transcend those with a direct genomic connotation and involve others that can modulate or be informative of the thermal responses. For instance, from an abiotic point of view, nutrient availability (Little et al., 2016), absorption (Wu et al., 2020), and soil interactions (Sedlacek et al., 2014) could act as enhancers or limiting factors of the adaptive responses. From a biotic perspective, among-ecotype differentiation (Cortés et al., 2012a,b, 2013; Blair et al., 2016), intrapopulation divergence (Cortés et al., 2011; Blair et al., 2012, 2018; Kelleher et al., 2012), and within-family variation (Galeano et al., 2012; Blair et al., 2013) could encourage or coerce adaptation. Population’s functioning, abundance, distribution, and diversity, as predicted from controlled experiments (Way and Oren, 2010; Elmendorf et al., 2012; Wolkovich et al., 2012; Andresen et al., 2016; Becklin et al., 2017; Singh et al., 2017), experimental evolution (Tenaillon et al., 2012; Mallard et al., 2018; Pfenninger and Foucault, 2020), biological monitoring (Walther et al., 2002; Franks et al., 2013; Wipf et al., 2013; Reichstein et al., 2014; Hällfors et al., 2020), and shifts observed in the fossil record (Alsos et al., 2009; Willis and MacDonald, 2011; Lyons et al., 2016; Bruelheide et al., 2018), can feed back on climate change (Pearson et al., 2013) and so be considered as drivers themselves. Regardless of the exact nature and extent of the data type, both GP and ML may offer suitable scenarios to merge diverse, and even conflicting, data sources in order to pinpoint emergent properties (Street et al., 2011) out of a complex system, as is thermal genomic adaptation. Therefore, a key guideline for new developments concerns a better coupling of GP and ML approaches. Until now, only a few works have relied on both methodologies, in the context of thermal adaptation in wheat landraces (Montesinos-Lopez et al., 2018, 2019), but have not gone beyond technical comparisons/recommendations, nor have designed integrated pipelines. Also, reconciling modern genomics with last-generation predictive inferences of the thermal adaptive potential and stochastic demographic modeling (Jenouvrier et al., 2009) is necessary. Open-access resources and data sharing platforms are as crucial in this effort as new integrated analytical pipelines. We are looking forward to seeing more cohesive (Beyer et al., 2020) and systematic studies and predictions across the rich and informative temporal spectrum (Kristensen et al., 2018) of past and future environmental variation (Franks et al., 2013). These efforts should be carried out through a wide range of spatial scales (Parmesan and Hanley, 2015; Way et al., 2015; Gonzalez et al., 2020) spanning contrasting ecosystems (Lenoir et al., 2020), microhabitats (Zellweger et al., 2020), and unrelated taxa, which together may already be keeping heritable adaptive trait differentiation valuable for long-term thermal responses and informative for conservation prioritizations (Barnosky et al., 2017; Elsen et al., 2020).

## Author Contributions

AC conceived this mini-review. FL-H collected the literature and prepared diagrams. DO-R compiled the historical climate data. AC wrote the first draft of the mini-review with further contributions from FL-H and DO-R. All authors contributed to the article and approved the submitted version.

## Conflict of Interest

The authors declare that the research was conducted in the absence of any commercial or financial relationships that could be construed as a potential conflict of interest.

## Supplementary Material

FIGURE S1 Past and future of thermal and CO_2_ variation, and their correlates with past biodiversity. **(A)** Temperature and richness of plant species (from pollen) for the Cenozoic Era (65 Mya – present). Temperature estimates (Supplementary Table S1) were computed by Hansen et al. (2013) using the original δ^18^O record from Zachos et al. (2008). Richness of plant species from pollen data (Supplementary Table S1) is based on 15 Neotropical stratigraphic sections inspected by Jaramillo et al. (2006). This profile goes from 65 to 20 Mya due to a lack of more recent suitable sampling records. **(B)** Projections of the near-surface temperature anomalies to 2,050 (Supplementary Table S2), which follow the CIMP5 RCP 8.5 scenario from the KNMI (http://climexp.knmi.nl/) repository averaged from an original 5-min resolution. Light gray shaded areas depict minimum and maximum estimates. **(C)** Atmospheric CO_2_ and richness of plant species (as in A) for the Cenozoic Era (65 Mya – present). CO_2_ records are an updated version (Supplementary Table S1) derived from Royer and Chernoff (2013), originally compiled by Beerling and Royer (2011). **(D)** Projected CO_2_ concentration (ppm) to 2,050 also follow the CIMP5 RCP 8.5 scenario, as in B (Supplementary Table S3).

TABLE S1 Dataset of temperature, atmospheric CO_2_, and richness of plant species for the Cenozoic Era (65 Mya – present for temperature and CO_2_, and 65–20 Mya for richness of plant species). Temperature estimates were computed by Hansen et al. (2013) from five-point running means of the original temporal resolution of the δ^18^O record from Zachos et al. (2008), a profile of surface low-magnesium calcitic fossils (including planktonic foraminifera, belemintes, brachiopods, and bivalves) that was lower during periods with warmer seawater. Atmospheric CO_2_ corresponds to an updated version from Royer and Chernoff (2013), originally compiled by Beerling and Royer (2011). Richness of plant species is based on pollen data from Jaramillo et al. (2006), who analyzed 1,530 samples from 15 stratigraphic sections in Colombia and Venezuela (Neotropics).

TABLE S2 Projections of thermal variation to 2,050. Simulation of Near-Surface Air Temperature Anomalies (°C) from 1,860 to 2,050 follow the CIMP5 RCP 8.5 scenario from the KNMI (http://climexp.knmi.nl/) database averaged from an original 5 min resolution. Minimum and maximum temperature estimates were generated by the coupled ACCESS *v.*1.0 model specifically designed for the CIMP5 project (Kowalczyk et al., 2013).

TABLE S3 Projections of CO_2_ concentration (ppm) to 2,050. Simulations follow the CIMP5 RCP 8.5 scenario from 1,860 to 2,050 available at KNMI (http://climexp.knmi.nl/) database averaged from an original 5 min resolution.
