# Supplementary figures and images for: Predicting Thermal Adaptation by Looking Into Populations’ Genomic Past
Source: Front Genet. 2020 Sep 25;11:564515. doi: 10.3389/fgene.2020.564515 (PMC7545011; doi:10.3389/fgene.2020.564515)

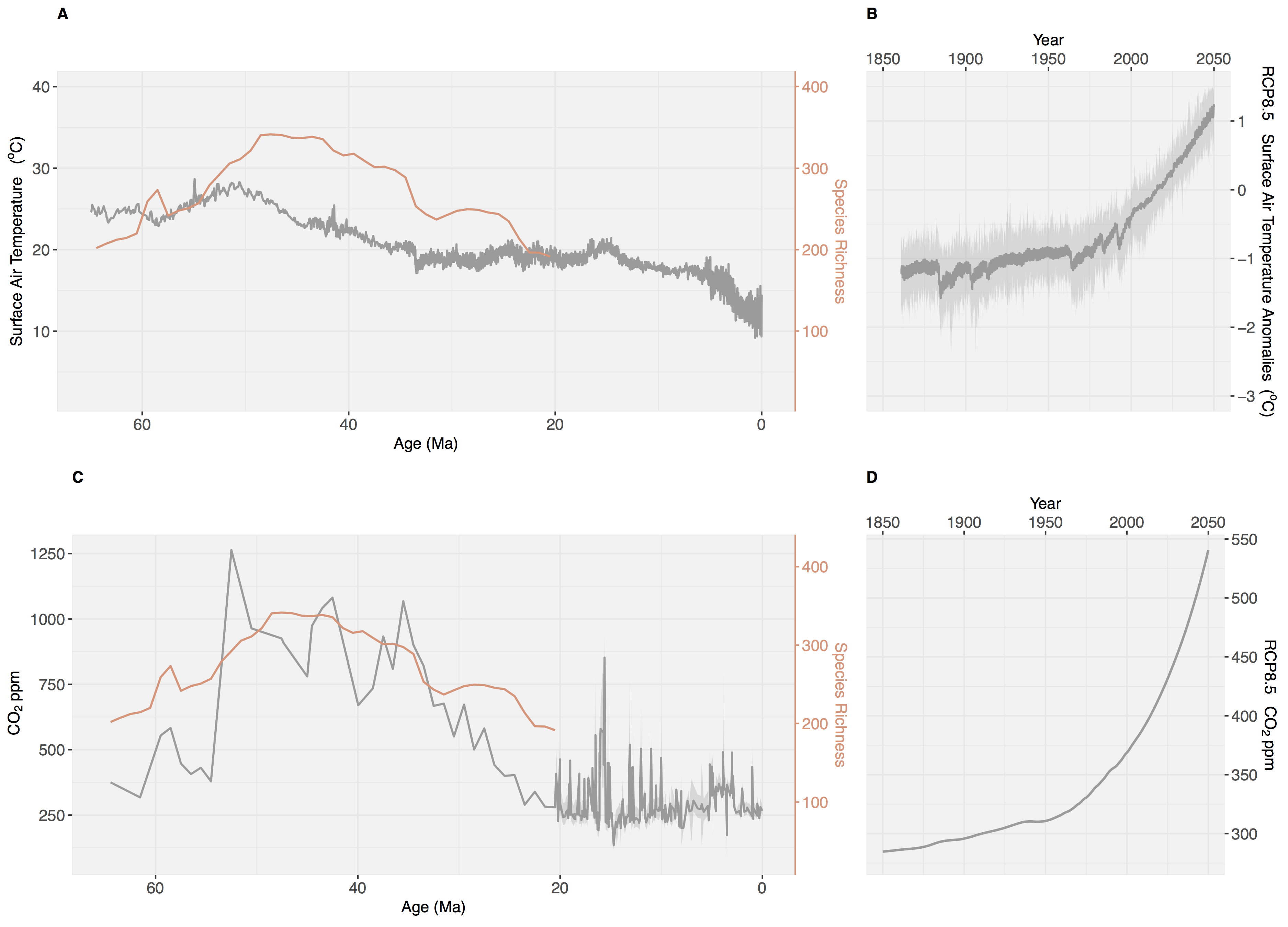

Supplement: FIGURE S1 — Past and future of thermal and CO2 variation, and their correlates with past biodiversity. (A) Temperature and richness of plant species (from pollen) for the Cenozoic Era (65 Mya – present). Temperature estimates (Supplementary Table S1) were computed by Hansen et al. (2013) using the original δ18O record from Zachos et al. (2008). Richness of plant species from pollen data (Supplementary Table S1) is based on 15 Neotropical stratigraphic sections inspected by Jaramillo et al. (2006). This profile goes from 65 to 20 Mya due to a lack of more recent suitable sampling records. (B) Projections of the near-surface temperature anomalies to 2,050 (Supplementary Table S2), which follow the CIMP5 RCP 8.5 scenario from the KNMI (http://climexp.knmi.nl/) repository averaged from an original 5-min resolution. Light gray shaded areas depict minimum and maximum estimates. (C) Atmospheric CO2 and richness of plant species (as in A) for the Cenozoic Era (65 Mya – present). CO2 records are an updated version (Supplementary Table S1) derived from Royer and Chernoff (2013), originally compiled by Beerling and Royer (2011). (D) Projected CO2 concentration (ppm) to 2,050 also follow the CIMP5 RCP 8.5 scenario, as in B (Supplementary Table S3). [file Image_1.tiff]
